# Everybody's Page

**Published:** 1888-03-24

**Authors:** 


### EVERYBODY'S PAGE.

LOOSE-FITTING clothing is—other things being equal—warmer than tight-fitting.

IN cases of burns caused by strong acids or alkalies, a good "ducking" should, if possible, be given at once. Failing that, douche or souse the patient with plenty of cold water.

DIPHTHERIA and hysteria are apt to cause paralysis of the vocal cords. This paralysis is, however, transient, or at least remediable, and is for the most part incomplete.

WHILE many children with no idea of music learn to speak soon, musical children are not infrequently slow to speak correctly.

BERTHOLD AUERBACH, the writer of so many charming German stories, said "An expressive countenance is our greatest enemy; therefore, with Heaven's help, I have made mine blank and dull."

DR. CONSTANTINE PAUL is of opinion that a scrofulous constitution is evidenced by the formation of a linear scar or slit-like opening where the lobule is pierced; but others have suggested that the weight of earrings may be sufficient to account for this peculiarity.

STEAM applied under moderate pressure for five minutes destroys the germs of most diseases. Boiling linen clothes for half-an-hour with a little soap or soda or common salt disinfects them thoroughly, if so managed that the heat reaches all parts.

OLD medical treatises contain many strange remedies for "worms in the ear," by which designation almost every kind of aural disease seems to have been known. Some old people still believe that their deafness is due to a blac k-beetle in the ear, and thousands of hapless earwigs have been sacrificed to the notion that they injure the organs of hearing.

SINGING, when well managed, is the very best means of strengthening the chest. But unfortunately there are some whose vocal attempts are painful to their neighbours. For such people the following exercise has been devised: Stand upright with the face looking rather upwards, and extend the arms straight in front of you, with the palms together. The arms are then to be slowly carried backwards horizontally, on a level with the shoulders as far as possible. While this movement is being made a deep breath is to be drawn. The arms are then to brought rapidly back and crossed upon the breast, so that the right hand lies on the left shoulder, and *vice versa*, a full expiration being then made. These movements ought to be repeated regularly for some minutes.

IN a Scottish law court there has recently been raised the question of the dishorning of cattle—is it a crime under the act for preventing cruelty to animals, or is it not? It has long been customary to cut off the horns of oxen near the base, and formerly the operation was looked upon as painless. But the principals of two veterinary colleges proved that it was very much the reverse. The cells of the core of the horn are lined with a sensitive membrane, which is continuous from the nostrils up to the points of the horns, and which is more sensitive at the base of the horn than elsewhere. Both gentlemen gave it as their opinion that the amputating of the horns is grossly cruel and unnecessary, and that tipping the horns with wood or iron balls would serve the desired purpose—of preventing vicious animals inflicting damage—quite as effectually. In spite of this evidence, however, the judge gave it as his opinion that the dishorning of cattle could not be looked upon as a crime. Nevertheless, we hope that it will soon be exchanged for a more merciful operation.

FOUR HUNDRED THOUSAND salmon ova are to be despatched to Tasmania for the purpose of stocking the rivers of that colony.

A "FIREPROOF STARCH," prepared with tungstate of soda, was introduced some years ago by Mr. Donald Nicoll. This starch, if used in the ordinary way, would render any fabric immersed in it non-inflammable.

SUNSTROKE might be more properly entitled "heat-stroke," for it does not of necessity depend upon the direct action of solar rays. Sunstroke may, indeed, occur when the sun is not shining; and at least one writer on the subject has asserted that "the direct influence of the sun has nothing whatever to do with its production."

MUSCULAR weakness in the structures that maintain the erect position of the spine may lead to curvature and other deformities of the back; and thus it is that the use of rigid corsets in young people has been so generally condemned by surgeons as a most efficient means for producing spinal ailments.

IT is a common thing to say that some garments are "warm" and others "cool." It is almost needless to say that articles of clothing possess neither warmth nor coldness in themselves. What is meant by a warm garment is one that is capable of retaining the natural heat of the body; while the cool garment, so-called, allows that heat to escape, and brings the surface of the body more immediately under the influence of the cooler atmosphere that surrounds it.

FOR those ladies who find, or think they find, all soap too harsh for their sensitive skins, steaming the face is recommended. Place a vessel containing hot water over a lighted spirit lamp (in order to keep the water at boiling point) and hold the face over it for ten minutes, gently pressing and kneading it all the time. The inventor of this method of cleansing the skin does not mention, but we think it right to state as a caution, that the face should not be held too near the boiling water. Steam when condensed sufficiently by contact with the air to be visible, is not dangerous, but pure steam can inflict a very severe burn.

MOST of us can call to mind examples of the curious mental effects produced by various sounds heard during sleep or a drowsy condition. Shakespeare tells us how, when Queen Mab drums in his ear, the soldier "starts and wakes, and, being thus frighted, swears a prayer or two." The vibrations of a pair of tweezers close to his ear as he slept, Mr. Maury found to occasion dreams of bells, the tocsin, and the revolution of 1848; and we have read of an ingenious lover who effected a change of sentiments in an unkind lady love by whispering his name in her ear when she was slumbering, and thus inducing in her a habit of happy dreams connected with him.

A GOOD many women wash their hair only at long intervals, asserting that the practice is injurious. This is quite a mistake. Women should shampoo their hair every two or three weeks; if they are exposed to much dust, every week or ten days. The head should be well rubbed with a lather of some good soap. All the soap should then be thoroughly rinsed out with warm water, and the hair and scalp should be thoroughly dried. Any evil resulting from washing the hair is caused by dressing it before it is dry. After washing, it is sometimes advisable to rub a little vaseline into the scalp, but not on the hair.

